# Perceptions, needs, and experiences of kinesiophobia management after total knee arthroplasty: a qualitative study from the perspectives of patients, caregivers, and healthcare providers

**DOI:** 10.3389/fpubh.2026.1861297

**Published:** 2026-07-03

**Authors:** Shuoni Ren, Ya'Nan Kan, Lin Chen, Xiaolan Zhang, Fuying Ye

**Affiliations:** 1 School of Nursing, Zhejiang Chinese Medical University, Hangzhou, Zhejiang, China; 2 Department of Orthopedics and Traumatology, The First Affiliated Hospital of Zhejiang Chinese Medical University (Zhejiang Provincial Hospital of Traditional Chinese Medicine), Hangzhou, Zhejiang, China; 3 Department of Nursing, The First Affiliated Hospital of Zhejiang Chinese Medical University (Zhejiang Provincial Hospital of Traditional Chinese Medicine), Hangzhou, Zhejiang, China; 4 Trade Union, The First Affiliated Hospital of Zhejiang Chinese Medical University (Zhejiang Provincial Hospital of Traditional Chinese Medicine), Hangzhou, Zhejiang, China

**Keywords:** cognition, experience, kinesiophobia, needs, qualitative research, total knee arthroplasty

## Abstract

**Aim:**

To explore the congruence and divergence in perceptions, needs, and experiences regarding kinesiophobia management among patients, caregivers, and healthcare providers following total knee arthroplasty.

**Methods:**

Using purposive sampling, semi-structured interviews were conducted with 12 healthcare providers, 12 patients following total knee arthroplasty, and their corresponding caregivers. Thematic analysis was used to analyze the interview data and to extract themes.

**Results:**

Three themes and nine subthemes were identified: perceptions (divergent views on the safety of functional exercise; redistribution of authority and co-construction of trust; overcoming kinesiophobia as contingent on perceived rehabilitation improvement; and the unseen weight of financial and emotional strain); experiences (empathy and mutual growth; from understanding to accompaniment; from frustration to perseverance); and needs (guidance on rehabilitation exercises; expectations regarding communication and collaboration).

**Conclusion:**

Although perceptions of kinesiophobia diverged among patients, caregivers, and healthcare providers, the three groups showed convergence in their rehabilitation needs and experiences. A collaborative intervention strategy centered on shared decision-making and integrating the perspectives of patients, caregivers, and healthcare providers is recommended to optimize rehabilitation management following total knee arthroplasty.

## 1 Introduction

Total knee arthroplasty (TKA) is a primary surgical intervention for end-stage knee osteoarthritis, and has been shown to significantly improve joint function and quality of life in affected patients (1). However, achieving favorable long-term rehabilitation outcomes critically depends on the standardization and continuity of functional exercise. Nevertheless, a previous study (2) as shown that approximately 24.4% of patients following TKA develop a fear of functional exercise, which significantly increases the risk of complications such as joint capsule adhesion and deep vein thrombosis (3). According to the fear-avoidance model (4), kinesiophobia is defined as a condition in which patients develop catastrophic cognitions due to previous painful experiences, which in turn leads to an excessive and irrational fear of functional exercise. Although the effectiveness of interventions such as exercise guidance for kinesiophobia has been preliminarily confirmed, the actual outcomes of these interventions remain suboptimal. This may be because kinesiophobia undermines patients’ motivation for rehabilitation after TKA while simultaneously leading to excessive concerns about the safety of physical activity during daily interactions. Meanwhile, caregivers are often required to assist in treatment decision-making, supervise daily activities, and provide emotional support, but due to insufficient knowledge and coping strategies regarding kinesiophobia, they tend to adopt conservative care strategies (5). However, although healthcare providers possess the professional competence to manage kinesiophobia after TKA, they have difficulty understanding the actual behavioral responses of patients and caregivers during rehabilitation. Cognitive discrepancies among patients, caregivers, and healthcare providers may lead to a disconnect between rehabilitation guidance and actual needs, thereby hindering the implementation of a patient-centered rehabilitation model. Thus, the management of kinesiophobia after TKA still urgently requires collaborative efforts among patients, caregivers, and healthcare providers (6, 7).

Most current studies on kinesiophobia after total knee arthroplasty are cross-sectional surveys or analyses of influencing factor (2), and the few qualitative studies available (5) are confined to a single perspective, such as that of patients alone. Furthermore, although previous research (8) has explored triadic interactions among patients, caregivers, and healthcare providers in chronic disease management, no qualitative study has yet investigated kinesiophobia after TKA from the triadic perspective. Without integrating the perspectives of the three stakeholder groups, interventions may fail to address the relational and contextual barriers that perpetuate kinesiophobia. Given the important role of interpersonal interactions and support systems in kinesiophobia management, this study aimed to explore the perceptions, needs, and experiences of patients, caregivers, and healthcare providers regarding kinesiophobia management after TKA, with the goal of providing a reference for developing targeted perioperative comprehensive management strategies for kinesiophobia.

## 2 Methods

### 2.1 Aim

To explore the perceptions, needs, and experiences of patients, caregivers, and healthcare providers regarding kinesiophobia management after TKA.

### 2.2 Design and participant recruitment

Purposive sampling was used to recruit patients who had undergone primary TKA and developed kinesiophobia after surgery, along with their primary caregivers and healthcare providers involved in perioperative management, at the Orthopedic Trauma Center of a tertiary hospital from November 2025 to January 2026. Sample size was determined based on the principle of thematic saturation. Recruitment ceased when no new themes emerged from two consecutive interviews, indicating that saturation had been reached. To ensure sample diversity, a maximum variation sampling strategy was used to select patients and their caregivers with different ages, genders, and other characteristics, as well as healthcare providers with varying years of clinical experience. The detailed inclusion and exclusion criteria are presented in Table 1.

**Table 1:** Inclusion and Exclusion Criteria for patients, cargivers,and healthcare providers.

Participants	Inclusion criteria	Exclusion criteria
Healthcare professionals (physicians, rehabilitation therapists, and nurses)	(1) Extensive clinical experience in post-TKA care	(1) Limited experience managing patients with kinesiophobia after TKA
	(2) Voluntary participation and ability to communicate effectively	(2) Declined to participant
Patients	(1) Age ≥ 18 years and provided written informed consent	(1) Severe mental illness, cognitive impairment, or language difficulties precluding effective participation in interviews
	(2) Underwent primary unilateral TKA(regardless of surgical approach or prothesis type)	(2) Severe comorbidities compromising study participation, including significant cardiac, pulmonary, hepatic, or renal insufficiency, or malignant tumors
	(3) Diagnosed with kinesiophobia (score ≥ 37on the Tampa Scale of Kinesiophobia, TSK-17) by the attending nurse based on clinical presentation and scale assessment at first ambulation post-surgery	(3) Declined participation or withdrew from the study
	(4) Voluntary participation and ability to communicate effectively	
Caregivers	(1) Age ≥ 18 years and provided written informed consent	(1) Severe physical illness, mental disorders, or language difficulties precluding effective participation in interviews
	(2) Identified as the primary caregiver for a patient meeting the eligibility criteria during the postoperative period	(2) Declined participation or withdrew from the study
	(3) Lives with patient or provide daily hands-on care	
	(4) Voluntary participation and ability to communicate effectively	

Each patient was matched with one corresponding primary caregiver. Healthcare providers were recruited based on their involvement in perioperative care, without requiring matching to individual patients.

### 2.3 Research methods

#### 2.3.1 Measures and instruments

Guided by the literature review and study objectives, data were collected using the following tool: (1) General information questionnaire, which was self-designed to obtain participants’ sociodemographic characteristics and clinical data. (2) Tampa Scale for Kinesiophobia (TSK-17): This scale consists of 17 items, each rated on a 4-point Likert scale (total score ranging from 17 to 68). A higher score indicates a greater degree of fear of movement. The Chinese version of the Tampa Scale for Kinesiophobia (TSK-17) translated by Hu et al. (9) was used in this study. This version has demonstrated good reliability and validity (Cronbach’s α =0.74). The cutoff score of the TSK-17 (total score ≥ 37), which had been clinically validated in previous TKA studies (2), was adopted to screen patients with kinesiophobia for participation in the interviews. (3) Semi-structured interview guide: The research team developed separate versions of the semi-structured interview guide for patients, caregivers, and healthcare providers. The guides were pilot-tested (with one patient, one caregiver, and one healthcare provider) and subsequently refined. The final interview guides are presented in Appendix 1.

#### 2.3.2 Data collection methods

The researcher completed TSK-17 screening and collected baseline data before the patient’s first functional exercise session after TKA, and conducted the interview half an hour after that session. The interview times for caregivers and healthcare providers were arranged on a flexible basis. Interviews were conducted in a private space within the inpatient ward, each lasting 20–40 min. All interviews were carried out by researchers who had received systematic training in qualitative research. To minimize social desirability bias, interviews were conducted by researchers who were not involved in the participants’ clinical care. Prior to each interview, participants were explicitly assured that their answers would not affect their treatment or relationship with healthcare providers, and that there were no right or wrong answers. All interviews were audio-recorded, with the researcher concurrently noting nonverbal cues (such as expressions, gestures, and emotional responses) and contextual details. During the interviews, the researcher followed the interview guide and used techniques such as probing, clarification, and summarization to encourage participants to elaborate on their experiences, while avoiding leading questions.

#### 2.3.3 Data management and analysis

Within 24 h after each interview, two trained researchers independently transcribed the audio recordings and organized the field notes. Thematic analysis, as proposed by Braun and Clarke (10), was used to analyze the transcribed data. The analysis process consisted of six phases: familiarization with the data, generating initial codes, searching for themes, reviewing themes, defining and naming themes, and producing the report. Data coding and management were performed using NVivo 14.0 software. In Initial coding was performed independently by two researchers, and disagreements were resolved through discussion. The initial codes were then grouped into candidate themes based on similarities and differences. These candidate themes were iteratively reviewed and refined through comparison across the three participant groups (patients, caregivers, and healthcare providers), ultimately leading to the development of higher-order themes. Data collection and analysis proceeded concurrently, with coding categories being continuously updated until thematic saturation was achieved. To specify the criteria for determining saturation, thematic saturation was assessed primarily using the combined dataset of all three participant groups as the core standard. Recruitment was terminated when two consecutive interviews (regardless of participant affiliation) yielded no new themes, indicating that overall saturation had been reached. Concurrently, the emergence of themes within each participant group (patients, caregivers, and healthcare providers) was separately monitored during analysis to ensure that no group-specific new themes were overlooked, thereby supporting the overall saturation judgment.

#### 2.3.4 Rigor

To ensure methodological rigor, the lead researcher engaged in continuous self-reflection to control for the potential influence of personal biases and preconceptions. Any discrepancies or unclear points in the transcribed texts were cross-checked by two researchers and, where necessary, confirmed with the interviewees. The research team held regular meetings to review the coding results and ensure the accuracy and consistency of theme extraction. During analysis, two researchers actively sought and discussed negative or discrepant cases that contradicted the main themes. Any identified divergent views were discussed by the team to determine whether to integrate them into existing themes or to develop new themes. All interview recordings and transcripts were systematically archived, and a complete audit trail was established. This study was reported in accordance with the COREQ checklist to ensure research transparency (11).

#### 2.3.5 Ethical considerations

This study involved interviews with human participants, including patients, caregivers, and healthcare providers. The study followed the basic principles of the Declaration of Helsinki, and the research protocol was reviewed and approved by the Ethics Committee of the First Affiliated Hospital of Zhejiang Chinese Medical University (approval No. 2025-KLS-602-01). Before the interviews, the researcher explained the purpose, significance, and ethical principles of the study to the participants, including the voluntary nature of participation and the right to withdraw at any time. All participants provided written informed consent before enrollment. The researchers ensured strict confidentiality of personal information, and all data were anonymized during transcription and analysis to prevent identification of individual participants.

## 3 Results

### 3.1 Characteristics of the study participants

A total of 36 participants completed semi-structured interviews, including 12 patients (*P1–P12*), 12 caregivers (*C1–C12*), and 12 healthcare providers (*H1–H12*). The mean age of patients after TKA was 69.2 ± 10.6 years, and the mean TSK-17 score was 46.5 ± 5.8, indicating the presence of significant kinesiophobia (4). The mean age of caregivers was 54.9 ± 10.8 years, with spouses accounting for 66.6% and children accounting for 33.3%. The mean age of healthcare providers was 34.8 ± 9.6 years, and their mean years of clinical experience was6.1 ± 3.8 years. Other demographic and clinical characteristics of the participants are presented in Table 2. The mean interview duration was 30 min (range: 20–40 min).

**Table 2:** Characteristics of patients with kinesiophobia following TKA, their caregivers, and attending healthcare professionals (*n* = 36).

	Characteristics		Participant (%)
Patient	Gender	Male	8
		Female	4
	Age	40~60 years	2
		60~80 years	8
		≥80 years	2
	Educational level	Junior high school or below	4
		High school	6
		College or above	2
	Marital status	Unmarried	1
		married	11
	Disease duration	<5 years	5
		≥5 years	7
	TSK-17 score	37~45 scores	4
		≥46 scores	8
Caregivers	Relationship to patient	Spouse	8
		Adult child	4
	Age (years)	40~49 years	5
		50~69 years	6
		≥70 years	1
Healthcare professionals	Position	Nurse	9
		Orthopedic surgeon	2
		Rehabilitation physician	1
	Years of experience	<2 years	2
		<5 years	5
		≥5 years	6
	Education level	Bachelor’s degree	7
		Master’s degree or above	5

### 3.2 Perceptions, needs, and experiences of patients, caregivers, and healthcare providers regarding the management of kinesiophobia after TKA

The themes derived regarding the perceptions, needs, and experiences of patients, caregivers, and healthcare providers concerning kinesiophobia management after TKA are presented in Table 3.

**Table 3:** Themes of perceptions, needs, and experiences regarding the management of kinesiophobia.

Themes	Sub-themes
Perceptions of kinesiophobia management after TKA	1. Divergent views on the safety of functional exercise
	2. Redistribution of authority and co-construction of trust
	3. Overcoming kinesiophobia is contingent on perceived rehabilitation improvement
	4. The unseen weight of financial and emotional strain
Experience of kinesiophobia management after TKA	1. Empathy and mutual growth
	2. From understanding to accompaniment
	3. From frustration to perseverance
Specific needs of kinesiophobia management after TKA	1. Guidance on rehabilitation exercises
	2. Expectations regarding communication and collaboration

#### 3.2.1 Perceptions of kinesiophobia management after TKA

##### 3.2.1.1 Divergent views on the safety of functional exercise

Patients often equate sensations such as “painlessness” and “immobility” with rehabilitation safety, and lack a clear understanding of the frequency and intensity of functional exercises. *“I think it is better to be conservative now, since I just had surgery a few days ago” (P5, postoperative day 3).* Caregivers prioritized preventing potential harm from exercise over achieving rehabilitation gains, leading them to hold conservative perceptions regarding the safety of rehabilitation activities and to limit or reduce the intensity and frequency of patients’ exercises. *“She is already so old, and her health was not good to begin with, so her recovery certainly will not be as fast as a younger person’s. It is fine just to do the movements roughly” (C3, spouse).* However, healthcare providers noted that patients and their caregivers sometimes held overly conservative attitudes toward functional exercise, failing to meet the actual intensity requirements for rehabilitation. *“In fact, patients and their families are overly conservative about the intensity of functional exercises. When functional exercise is performed properly in accordance with rehabilitation guidelines and biomechanical principles, it is not a painful process” (H10, orthopedic surgeon).*

##### 3.2.1.2 Redistribution of authority and co-construction of trust

Some patients initially relied on caregivers’ advice but began to doubt it when caregivers could not answer questions about pain or progress. After interacting with healthcare providers, they placed greater trust in professional guidance than in family recommendations. *“I listen to my children and do whatever they tell me to do. But later I found that the nurse’s instructions made more sense than what my family said” (P6, 68 years).* Caregivers reported that inconsistent or unclear guidance from different healthcare providers left them with decreased trust but no initiative to ask questions. They felt helpless in decision-making and observed increased fear and avoidance of functional exercises in themselves and the patients. *“I remember the doctor previously advised him to move less, but did not say when he could start moving. I had questions but was afraid to ask” (C2, patient’s spouse).* Healthcare providers reported using consistent communication, empathetic listening, and clear explanations of rehabilitation rationales to build trust with patients. They stated that such trust was important for patients to accept and adhere to professional recommendations regarding kinesiophobia management. *“I think the most important thing is to build trust with the patient, so that they will listen to whatever advice I give” (H2, nurse).*

##### 3.2.1.3 Overcoming kinesiophobia is contingent on perceived rehabilitation improvement

In the early rehabilitation phase after TKA, patients often feel fearful about exercising and perform prescribed movements only perfunctorily or tentatively. As they gradually adapt to the movements and experience improvements in physical sensations, their fear of joint loading progressively subsides. *“I think it is not as scary as I imagined; the hardest part is really taking the first step” (P3, female).* Caregivers reported that they had higher levels of engagement in rehabilitation activities when patients achieved better functional recovery. Many caregivers noted that once patients could perform certain movements without pain or without assistance, they no longer felt it necessary to limit the patients’ exercise. *“I think she just does not believe in herself; she walks much better now than before” (C6, daughter) According to healthcare providers, patients’ shift from passivity to active participation was motivated more by tangible functional gains they experienced themselves than by verbal reassurances not to fear movement. “They may show behaviors or emotions indicating low satisfaction, but once they see results, they will actively cooperate with you” (H11, rehabilitation physician).*

##### 3.2.1.4 The unseen weight of financial and emotional strain

Several young patients who were temporarily unable to work reported a loss of adult identity, along with guilt toward caregivers and an urgent wish to recover quickly. They also reported that being unable to work was a major source of distress, whereas physical discomfort was not their primary concern. “*Actually, seeing my parents busy every day makes me feel really guilty, so I just want to get better quickly” (P10, 32 years old).* Caregivers reported that they were more concerned about improvements in patients’ condition than about economic pressures. They worried that rehabilitation instructions were too professional for them to judge exercise correctness, and expressed a need for nurses to supervise daily exercises and provide regular feedback. *“I really wish the healthcare staff could explain more to us. Could you please go and talk to them?” (C4, patient’s spouse).* Healthcare providers understood the needs of patients and caregivers but felt fatigued from repeatedly explaining basic exercise safety. They hoped that patients and caregivers would actively learn key rehabilitation knowledge during hospitalization to reduce dependence on repeated instruction after discharge. *“Actually, I would prefer that patients and caregivers remember some of the knowledge we explain, rather than us having to repeat the same content over and over” (H6, nurse).*

#### 3.2.2 Experience of kinesiophobia management after TKA

##### 3.2.2.1 Empathy and mutual growth

Patients gradually recognized that the benefits of overcoming kinesiophobia were more significant than simply reducing discomfort. They reported using assistive devices, setting small achievable goals for themselves, and tolerating pain to persist with functional exercises despite the accompanying discomfort. *“Before every step, I think about whether it will hurt, but then I think about being able to walk normally in the future. So I grit my teeth and slowly step forward with the support of my walker” (P4, female). C*aregivers initially felt confused about patients’ fear of exercise after witnessing reactions such as trembling. However, after in-depth communication with patients, they came to understand the rationality of this fear, and actively used physical comfort and verbal encouragement to alleviate patients’ emotional distress. *“I can tell when she is feeling down. When that happens, I pat her on the shoulder and tell her to take it easy, and then her mood improves a lot” (C3, patient’s spouse).* Healthcare providers reported that they initially followed standardized rehabilitation protocols. However, after observing avoidance or nonadherence in patients due to fear, they began to actively listen to patient concerns and adjust exercise intensity or sequences based on the comfort levels patients themselves reported. *“A patient told me that he now understands that moving more actually leads to faster recovery. Seeing his progress makes me very happy” (H7, nurse).*

##### 3.2.2.2 From understanding to accompaniment

Patients undergoing TKA desired not only soothing touch and reassuring words from caregivers and providers, but also recognition of their small incremental progress. They described this type of companionship during difficult exercises as something that made them feel more confident in their recovery. *“I really wish the rehabilitation therapist could stay beside me a little longer. When he watches me exercise, I dare to move; otherwise, I’m always afraid I might do it wrong” (P1, female).* Caregivers reported that they initially attempted to prevent any activity that might cause harm, and later actively encouraged patients to exercise within safe limits. They also stated that their relationship with patients was previously tense, but became collaborative in daily rehabilitation after sharing the experience of managing kinesiophobia. “Before, I was always afraid that she might fall. Now I understand that accompanying her during exercise is more helpful than restricting her. When she walks, I support her by her side, and that gives her peace of mind” (C6, daughter) At specific milestones such as first ambulation or achieving target knee flexion angle, healthcare providers used step-by-step demonstrations and immediate brief verbal affirmations in response to patients’ actions. Healthcare providers observed that patients who gained such first-hand successful experiences showed confidence in performing functional exercises. *“The first time getting out of bed is the scariest for patients. We support them and teach them how to exert force. Once they take that first step, things become much easier” (H11, rehabilitation therapist).*

##### 3.2.2.3 From frustration to perseverance

Some patients viewed each exercise session as an investment in their future mobility, accepted the accompanying pain as a worthwhile cost, and continued to persist with functional exercises. They explained that their motivation for persistence came not from immediate effects, but from a concern that giving up would lead to regression. *“It’s been over a month of exercising, but my knee is still swollen and walking still hurts. So I started wondering whether the surgery wasn’t done properly or whether I should not have exercised so much” (P7, 62 years old).* Caregivers initially felt frustrated by patients’ slow rehabilitation progress. However, patients’ strong desire to resume daily life moved them deeply, leading them to overcome their frustration and persist in accompanying patients during exercises. *“I saw on social media that walking more leads to faster recovery, so I pushed him to walk more. Now I make sure to accompany him in his exercises every day” (C4, spouse).* In response to patients’ and caregivers’ repeated cognitive and emotional fluctuations, healthcare providers did not focus solely on technical adjustments. Instead, they chose to stay with them, patiently listening to their complaints and unease, and gradually shifted their communication focus from early relief of pain-related fear to later guidance on appropriate activity beliefs. *“Initially, we help patients relieve pain and overcome their fear of moving, and later we teach them how to protect their joints in daily life and avoid the risks associated with incorrect postures” (H5, orthopedic surgeon).*

#### 3.2.3 Specific needs of kinesiophobia management after TKA

##### 3.2.3.1 Guidance on rehabilitation exercises

Patients worried that without professional supervision at home, they might perform exercises incorrectly, leading to reinjury or ineffective rehabilitation. Consequently, they hoped that caregivers would act as home-based supervisors to confirm correct performance and relay concerns to healthcare providers when needed. *“I actually hope my family can learn some knowledge about functional exercises, because there are some movements I cannot complete on my own after returning home” (P1, 37 years old).* Caregivers reported that professional terminology and static diagrams in written or verbal instructions were difficult to translate into daily practice, and they often could not judge whether patients performed movements correctly. They desired brief video demonstrations aligned with rehabilitation stages, along with easily accessible checklists of key movement points. *“If there were an app that could let us contact our primary doctor at any time, that would give us peace of mind” (C7, patient’s daughter).* Some healthcare providers reported lacking standardized tools and quantitative criteria for kinesiophobia assessment, relying instead on clinical experience. They desired condition-specific scales, clear procedural guidance, and simple devices to determine severity and underlying causes. *“We want to help patients overcome their fear, but with no unified assessment criteria and outdated, monotonous equipment, sometimes even we ourselves are unsure what the best way to exercise is” (H9, nurse).*

##### 3.2.3.2 Expectations regarding communication and collaboration

Patients after TKA expected healthcare providers to fully consider their individual exercise preferences and pain tolerance thresholds, and to adopt two-way negotiation rather than unilateral instruction in rehabilitation decision-making. Patients believed that this communication-based collaborative relationship could enhance their willingness to participate and treatment adherence. *“I told the nurse I preferred sitting over standing because it felt safer, so they arranged more seated exercises for me and that made my daily practice much less difficult” (P4, male).* Caregivers reported that when patients doubted or resisted exercise, their own persuasion often failed. They expected healthcare providers to consistently reinforce the same rehabilitation messages with professional authority, shifting patients from resistance to active engagement. *“I used to encourage her to exercise, but she always found me annoying; then later the doctor said exactly the same thing during ward rounds, and she took it in” (C5, patient’s spouse).* When patients questioned junior providers’ competence, healthcare providers sought endorsement from higher-authority clinicians. They expected attending physicians to reinforce kinesiophobia messages during preoperative talks or morning rounds, thereby strengthening patient-provider trust. *“Patients tend to ask the orthopedic surgeons for confirmation because they believe only what the attending physician says counts, so we adjusted our communication approach by having the chief physician emphasize the key points during rounds, which makes patients feel much more reassured” (H3, 3 years of experience).*

## 4 Discussion

This study is the first to explore the perceptions, needs, and experiences regarding the management of kinesiophobia after total knee arthroplasty (TKA) from the tripartite perspectives of patients, caregivers, and healthcare professionals. The results revealed significant differences in perceptions of kinesiophobia among the three parties, yet their rehabilitation needs and experiences tended to converge. This may be attributed to the fact that patients’ fear of functional exercise arises from physiological sensations, caregivers perceive kinesiophobia indirectly through patients’ subjective reports, whereas healthcare professionals’ understanding is more grounded in clinical guidelines. Moreover, patients’ strong desire for recovery, caregivers’ emotional compromise, and healthcare professionals’ strategic adjustment collectively facilitates the convergence of the three parties in terms of rehabilitation needs and experiences.

Patients undergoing TKA often hold overly optimistic expectations for rehabilitation, which predisposes them to resistant behaviors when experiencing mild discomfort during early exercise, a pattern that is consistent with the mechanism underlying kinesiophobia (12). The underlying reason for this behavior lies in patients’ uncertainty regarding improvement in rehabilitation outcomes, which in turn weakens their accurate perception of risk boundaries. Cai et al. (13) also found that this avoidant cognitive bias is more prevalent among older adults with low rehabilitation motivation and high pain sensitivity, manifesting as excessive concern regarding activity safety. Furthermore, the covert nature of kinesiophobia, as a dynamic psychological variable, hinders caregivers from accurately recognizing it and engaging in effective communication with patients. However, caregivers’ responses to kinesiophobia are largely confined to emotional compromise, making it difficult for them to balance emotional support and scientific intervention. This cognitive limitation not only fails to correct patients’ avoidance tendencies but also rationalizes their avoidant behaviors through acquiescence. In a social context that defers to authority, the guiding role of healthcare professionals as authoritative figures helps to disrupt the aforementioned vicious cycle. The trust of both patients and caregivers in medical authority forms the foundation of that authority, with caregivers playing a key role in guiding patients to adhere to professional instructions (14). Li et al. (15) also noted that if the authority of medical decision-making is disconnected from the family support system, it can hardly play an effective guiding role. This suggests that, despite significant differences in the three parties’ perceptions of kinesiophobia, patients can still dynamically adjust their attitudes toward functional exercise during rehabilitation interactions through the mediating role of caregivers and the guidance of medical authority. Unlike previous studies that focused solely on the individual patient (16), the above-mentioned cognitive differences and interactive adjustment suggest a potential direction for integrating the perspectives of both patients and caregivers into rehabilitation manuals and training resources. It is recommended that preoperatively, patients be helped to establish reasonable recovery expectations, while caregivers are also enabled to understand the functional exercise protocol and safety boundaries as secondary prevention information, thereby ensuring that both parties receive consistent and authoritative content. Additionally, it is recommended that during postoperative rehabilitation, healthcare professionals dynamically integrate staged functional exercises with guidance on daily living, proactively identify patients’ uncertainty regarding rehabilitation outcomes, and transform caregivers’ emotional compromise into a collaborative basis for scientific intervention, thereby helping caregivers acquire the core skill of guiding patients to control joint activity intensity. As demonstrated by Bailey et al. (17), this collaborative cognitive transmission helps reduce fear of functional exercise, thereby bridging cognitive differences and eliminating multi-party communication barriers.

In patients undergoing TKA, adherence to postoperative rehabilitation exercises depends partly on situational factors, including therapist accompaniment. More importantly, it relies on whether, through repeated practice, patients can internalize the cognition that “exercise facilitates recovery” as a motivation for active participation (18). The key to this process is enabling patients to gain a sense of control over their rehabilitation progress through autonomous practice, thereby facilitating the transition from passive compliance to active participation. Although patients’ autonomous practice is essential, caregivers’ verbal encouragement can similarly modulate their motivation to exercise. This bidirectional interaction points to the potential for caregivers to take on both emotional support and cognitive guidance, whereas improving the kinesiophobia experience depends more on promoting constructive patient–caregiver interactions (19, 20). Unlike patients and caregivers, healthcare professionals face unique practical challenges in managing kinesiophobia. Constrained by workload and other limitations, healthcare professionals may not fully address the psychological needs of patients and caregivers. This forces them to prioritize strategic adjustments in their interventions under restricted conditions, and they gradually come to recognize that flexible responses are more effective than passive ones in gaining trust and cooperation (21). In the process of dynamically adjusting intervention strategies, healthcare professionals become more attuned to changes in the rehabilitation progress of both patients and caregivers, which is key to driving the convergence of experiences among the three parties. Our findings on the tripartite experience of kinesiophobia reveal that patients reframe exercise as an investment in future mobility, caregivers shift from protective restriction to active companionship, and healthcare professionals transition from standardized protocols to flexible adjustments centered on patient feedback. This suggests that the effectiveness of kinesiophobia management depends on creating experiences in which all three parties jointly witness gradual success, rather than unilaterally pursuing fear elimination. To achieve such shared experiences, future interventions for kinesiophobia may consider using remote intelligent monitoring technology to capture patients’ real-time status, thereby extending their sense of safety in the rehabilitation environment from the hospital to the home setting (22, 23). In addition, the real-time monitoring and emergency call functions of smart devices are expected to facilitate timely assistance in acute situations, thereby alleviating patients’ anxiety and distress (24, 25). Even under the influence of traditional values such as “patient-centeredness,” rehabilitation decisions are still primarily dominated by family members, including caregivers (26, 27). This is consistent with the observation by Bragstad et al. (28) that limited patient participation is a key factor constraining service effectiveness. Therefore, clinical interventions urgently need to enhance patients’ autonomy in rehabilitation exercise and shift the caregiver’s role from “decision-maker” to “supporter.” This aligns with the emphasis by Quinn et al. (29) on positioning patients as decision-making subjects, while more strongly underscoring the transfer of rehabilitation leadership back to the patient. This redistribution of rehabilitation authority can enhance psychological acceptance in patients with kinesiophobia and facilitate their transition from passive acceptance to active participation in rehabilitation exercises.

The management of kinesiophobia rehabilitation in China remains at a developmental stage, with only a few orthopedic centers capable of providing systematic rehabilitation assessment and guidance (30). This has resulted in a significant gap between patients’ need for continued, individualized rehabilitation guidance after discharge and the availability of medical resources (31). Meanwhile, although patients, caregivers, and healthcare professionals differ in their needs regarding kinesiophobia management after TKA, they all expect that assessment and guidance be available throughout the entire rehabilitation process. The above findings suggest that the current rehabilitation support system is relatively weak in the continuity of care, whereas the implementation of enhanced recovery after surgery (ERAS) may further restrict opportunities for patient–provider interaction. Against this background, the present study highlights that explicit role allocation between patients and healthcare providers is crucial for the successful delivery of rehabilitation guidance for kinesiophobia (32). Specifically, orthopedic surgeons should assume responsibility for delivering knowledge about kinesiophobia and communicating rehabilitation expectations. Rehabilitation therapists should provide stepwise skill guidance. Nurses should collaborate with caregivers to reinforce patients’ awareness of active participation. Notably, the effective implementation of the above technical pathway depends not only on caregivers’ accurate documentation and timely feedback regarding patients’ kinesiophobia rehabilitation progress, but also on the professional competence and communication skills of healthcare providers (33, 34). Among these professionals, nurses, as the core providers of basic care and clinical observation, directly influence the timeliness and effectiveness of kinesiophobia interventions through their assessment and feedback capabilities. This suggests that strengthening nurses’ training in standardized assessment and intervention tools for kinesiophobia, along with enhancing simulation-based training addressing patients’ physiological and psychological characteristics, holds potential value for improving their clinical competence in kinesiophobia management. However, relying solely on human resource optimization may not be sufficient to fully address the discontinuity between inpatient and outpatient rehabilitation support, and technological approaches are still needed to bridge this gap. Based on the needs assessment of the three parties in this study, integrating diversified health education strategies with mobile health technologies may be a potential direction to mitigate this discontinuity (35). Future efforts could be directed toward designing patient-centered self-management support tools for kinesiophobia, such as embedding staged rehabilitation goals and an activity progress self-assessment module into a mini-program. This design may help patients develop realistic rehabilitation expectations and may enhance their sense of control over their own rehabilitation process. Concurrently, supporting educational modules for caregivers could be developed. These modules may cover safety boundaries for appropriate activity assistance and communication skills for emotional support and behavioral guidance, which may help caregivers shift from passive accommodation to active collaboration. Based on the above measures, future studies could further examine the feasibility of evidence-based out-of-hospital management tools for kinesiophobia in bridging in-hospital guidance and home-based rehabilitation, including the application of standardized messaging and real-time feedback mechanisms. Additionally, the potential application of wearable devices for the continuous collection of patient activity levels and self-reported pain data warrants further exploration. Such data may help identify risk behaviors and send alerts to healthcare providers, thereby providing supporting evidence for individualized interventions under limited healthcare resources (36). It should be noted that the above recommendations are grounded in the experiences of the study participants and are intended to provide directional guidance for the development of future interventions. Their feasibility requires further exploration in subsequent research.

## 5 Limitation and future directions

Although this study ensured methodological rigor through strategies such as systematic thematic analysis and audit trails, the following limitations should be acknowledged. First, data were collected during the early postoperative period, when patients’ accounts of their kinesiophobia experiences may have been influenced by factors such as acute pain, emotional vulnerability, and recent experiences, and when they remained in close interaction with caregivers and healthcare providers. Although this interview context contributed to the richness of the data, it may have also discouraged participants from expressing negative experiences or divergent views. Although appropriate measures were taken to reduce social desirability bias, this bias could not be completely eliminated. Future studies are advised to adopt longitudinal designs, including post-discharge or multi-timepoint interviews, to differentiate between transient and persistent kinesiophobia and to examine how interview context may shape participant narratives. However, differences in the availability and quality of rehabilitation resources across geographical regions may contribute to heterogeneity in the presentation of kinesiophobia. Furthermore, the participants in this study were predominantly highly motivated and willing to express themselves. Although maximum variation sampling was used to enhance sample heterogeneity, the study population may still not fully represent patients with poor rehabilitation outcomes or low adherence. Therefore, cautious transferability of the findings to similar clinical settings is warranted. Future research should include multicenter stratified surveys and pay particular attention to these under-represented subgroups to achieve a more comprehensive understanding of kinesiophobia management after total knee arthroplasty.

## 6 Conclusion

The study revealed a pattern of divergent perceptions yet convergent needs and experiences among patients, caregivers, and healthcare providers regarding kinesiophobia management following TKA. This finding suggests that the experiential fear of patients and caregivers and the guideline-driven management of healthcare providers may be complementary rather than oppositional. Although this study does not yet provide a mature basis for an integrated intervention model, it points to a future direction that takes shared rehabilitation needs and emotional experiences as an entry point and strategically addresses cognitive discrepancies. This offers potential possibilities for refining collaborative kinesiophobia management strategies and facilitating their clinical translation.

## Data Availability

The raw data supporting the conclusions of this article will be made available by the authors, without undue reservation.
