# Supplementary material for: Perceptions, needs, and experiences of kinesiophobia management after total knee arthroplasty: a qualitative study from the perspectives of patients, caregivers, and healthcare providers
Source: Front Public Health. 2026 Jul 3;14:1861297. doi: 10.3389/fpubh.2026.1861297 (PMC13375708; doi:10.3389/fpubh.2026.1861297)
Supplement: Supplementary file 1 [file Table_1.docx]

**1. Interview Guide for Patients**

**A. Perceptions and Understanding of Kinesiophobia**

How long has it been since you underwent total knee arthroplasty?

Conceptual understanding: How did you first become aware of the concept of "kinesiophobia"? What does this term mean to you? How would you explain it to your family members?

Self assessment: How would you rate the severity of your current kinesiophobia? (e.g., mild, moderate, severe; or on a scale of 0 to 10)

Core impact: In what specific ways does this fear influence your attempts to engage in or adhere to rehabilitation exercises? (Please describe from both physical sensations and psychological perspectives)

Underlying fears: What are your deepest concerns or fears regarding postoperative exercise?

Attribution and typology: What do you believe contributes to your fear of physical activity? (e.g., fear of pain, fear of falling, concerns about prosthetic complications, etc.) Do you think the reasons for fear may vary among different patients?

Pain perception: How do you perceive the pain experienced during postoperative rehabilitation? How does this pain affect your mood and confidence in recovery?

Perceived social support: When you feel fearful or reluctant to engage in physical activity, do you prefer to face these feelings alone or with companionship and support? What is your current situation in this regard?

**B. Practice, Management, and Challenges of Functional Exercise**

Exercise adherence: Are you currently adhering to your rehabilitation exercises? What is the approximate frequency and duration of your exercise sessions?

Protocol adherence: Where did your exercise regimen originate (e.g., from physicians, therapists, family members, or self-directed)? Do you strictly follow the prescribed plan? Why or why not?

Behaviors and decision making: When feelings of fear are particularly intense, what do you typically do? (e.g., persevere despite discomfort, reduce exercise intensity, discontinue immediately, seek reassurance, etc.) What factors influence your decision in such situations?

Facilitators and barriers: What factors are most helpful in enabling you to overcome fear and complete your exercises? (e.g., family encouragement, observable progress, professional guidance, etc.) Conversely, what factors pose the greatest obstacles? (e.g., increased pain, exercising alone, lack of understanding regarding the purpose of exercises, etc.)

Self efficacy: How confident are you in your ability to manage this fear and successfully engage in functional exercises to complete your rehabilitation training

**C. Patient Provider Communication, Medical Advice, and Support Needs**

Information reception and comprehension: How did healthcare providers explain the importance and methods of postoperative exercise to you? Were their explanations clear and helpful to you?

Conflicting advice and decision making: How do you respond when exercise instructions from physicians evoke significant fear or discomfort? Do you ever modify the prescribed exercise plan on your own?

Support needs: What types of assistance do you perceive as most necessary in managing your fear of physical activity? (e.g., more detailed exercise guidance, pain management strategies, psychological counseling, etc.) In what specific ways do you wish your family members or caregivers could support you?

Perceptions of responsibility: In your view, what responsibilities should physicians, yourself, and your family members respectively assume in managing postoperative kinesiophobia and facilitating rehabilitation?

**D. Open Ended Questions**

Is there anything else you would like to share regarding your experiences or feelings about postoperative rehabilitation and managing fear of activity?

Do you have any specific suggestions or expectations for the medical team or your family members?

**2. Interview Guide for caregivers**

**A. Core Perceptions and Experiences of Caregivers(This section aims to explore caregivers' fundamental understanding, observations, and personal experiences regarding kinesiophobia.)**

Conceptual understanding: How did you first become aware of the concept of "kinesiophobia"? (e.g., through healthcare providers, educational materials, or personal observation) How do you understand this term?

Symptom recognition: What specific behaviors, emotional responses, or verbal expressions have you observed in the patient that led you to believe he or she may be experiencing kinesiophobia? (e.g., refusal to exercise, excessive worry, atypical descriptions of pain, etc.)

Attribution analysis: From your perspective, what factors may contribute to or exacerbate the patient's kinesiophobia? (e.g., fear of pain, distrust of surgical outcomes, prior experiences, psychological factors, etc.)

Information reception: How have healthcare providers explained or communicated the patient's kinesiophobia to you? In what ways have these explanations facilitated your understanding and management of the condition, and what limitations have you identified?

**B. Role Practice and Management Challenges of Caregivers**(This section focuses on caregivers' specific behaviors, strategies, and challenges encountered in daily caregiving.)

Exercise supervision role: What role do you typically assume when assisting or encouraging the patient to perform rehabilitation exercises (e.g., encourager, supervisor, exercise partner)? Could you describe a successful or unsuccessful experience in this regard?

Behavioral coping strategies: What specific measures do you usually take when the patient strongly resists exercise due to fear (e.g., communication and persuasion, temporarily discontinuing exercise, seeking external assistance)? Do you ever modify the exercise plan (e.g., frequency, duration) based on the patient's level of fear? If so, how do you adjust it?

Facilitators and barriers: In your experience of helping the patient cope with kinesiophobia, what factors do you perceive as most effective (e.g., observable patient progress, professional guidance, family support)? Conversely, what factors pose the greatest obstacles (e.g., insufficient personal nursing knowledge, patient's emotional fluctuations, lack of immediate support)?

**C. Internal Experiences and Support Needs of Caregivers(This section focuses on caregivers' psychological burden, personal beliefs, and perceived needs regarding support systems.)**

Personal beliefs and stress: What are your primary sources of stress or confusion when confronting the patient's kinesiophobia? How do you perceive your role and responsibility in the patient's rehabilitation process?

Empowerment and motivation: Based on your experience, what approaches do you believe may be most effective in motivating the patient to initiate and adhere to exercise? (e.g., setting small goals, role model encouragement, gamification of exercises, etc.)

Explicit support needs: What types of support or resources would you most desire to better assist the patient in managing kinesiophobia? (e.g., clearer and more understandable exercise guidance, techniques for addressing patient emotions, accessible professional consultation channels, psychological support services for caregivers, etc.)

**D. Comprehension and Implementation of Medical Advice** **(This section aims to explore caregivers' interpretation and execution of professional rehabilitation protocols, which is critical for evaluating intervention effectiveness.)**

Protocol comprehension and modification: How do you understand the rehabilitation exercise plan provided by healthcare professionals? In practice, have you felt the need to modify it? If so, for what reasons?

Decision making dilemmas: How do you weigh and decide when there is a direct conflict between the physician's exercise recommendations and the patient's fear related resistance?

Feedback and communication: By what criteria do you typically judge whether an exercise is "safe" or "effective"? How do you communicate the patient's actual responses, including their fear related reactions, to the medical team?

**3. Interview Guide for caregivers for Healthcare Providers**

**A. Nurses' Role Perception and Clinical Practice**

**(This section aims to clarify nurses' self positioning, daily responsibilities, and practical experiences in kinesiophobia management.)**

Role identification: Please briefly describe your clinical role and primary scope of practice in the orthopedic or joint surgery department.

Scope of responsibilities: What specific nursing tasks do you undertake in the management of kinesiophobia among patients following total knee arthroplasty? (e.g., assessment, education, exercise guidance, psychological support, etc.)

Practical strategies: In your clinical experience, which nursing or intervention approaches have proven particularly effective in alleviating kinesiophobia? (e.g., staged goal setting, sharing successful cases, mindfulness relaxation techniques, etc.)

Practice objectives: In your view, what should be the primary goals of nursing management for kinesiophobia? (e.g., reducing fear, enhancing exercise adherence, preventing complications, etc.)

**B. Nurses' Perceptions and Attribution of Kinesiophobia**

**(This section explores nurses' understanding of the kinesiophobia phenomenon, its underlying causes, and factors influencing its presentation.)**

Conceptual understanding: How do you understand the concept of "kinesiophobia"? In your view, how does it differ from ordinary postoperative pain or general fear of rehabilitation?

Attribution analysis: Based on your clinical experience, what do you perceive as the primary factors contributing to the development of kinesiophobia in patients following total knee arthroplasty? (e.g., pain experiences, concerns about surgical outcomes, individual psychological characteristics, etc.)

Identifying manifestations: What typical psychological states and behavioral patterns do patients with kinesiophobia commonly exhibit? (e.g., excessive anxiety in emotional responses, avoidance behaviors regarding exercise, repeatedly seeking reassurance through verbal expressions, etc.)

Stage specific characteristics: In your experience, how do the manifestations of kinesiophobia and the focus of its management differ across various phases of patient recovery (e.g., acute postoperative phase, intermediate rehabilitation phase, home based recovery phase)?

**C. Facilitators, Barriers, and Support Needs in Kinesiophobia Management**

**(This section focuses on the contextual factors and perceived needs that influence nurses' capacity to effectively manage kinesiophobia.)**

Facilitators and barriers: In your clinical practice, what factors most facilitate your ability to effectively manage kinesiophobia? (e.g., adequate time for patient education, clear nursing care pathways, team support, etc.) Conversely, what factors constitute the primary obstacles? (e.g., insufficient nursing staff, lack of cooperation from patients or family members, absence of systematic training, etc.)

Core support needs: What types of support or resources do you personally require most to better manage kinesiophobia? (e.g., more practical assessment tools, standardized communication scripts, interdisciplinary collaboration protocols, etc.)

Areas for improvement: In your view, what aspects of current clinical kinesiophobia management have the greatest potential for improvement? What novel interventions or support tools would you anticipate being most beneficial?

**D. Multidisciplinary Collaboration and Role Boundaries**

**(This section aims to delineate the patterns of team collaboration and role delineation from the perspective of nurses.)**

Perceptions of responsibilities: In your view, what core responsibilities should physicians, rehabilitation therapists, nurses, patients, and family members respectively assume in the management of kinesiophobia? What do you perceive as the unique contribution of nurses in this context?

Current collaboration and ideal model: How is multidisciplinary collaboration currently conducted in your department to jointly manage kinesiophobia? What would you consider to be an ideal model of collaboration?

Considerations for special populations: Do you emphasize or modify aspects of your management strategies for kinesiophobia when working with patients of different age groups (e.g., older adults versus middle aged and younger patients)?
